# Neuropsychiatric symptoms cluster as primary drivers of Long COVID complexity: a South Texas retrospective cohort study

**DOI:** 10.3389/fneur.2025.1612489

**Published:** 2025-07-23

**Authors:** Anne Marie Wells, Summer Rolin, Barbara Robles-Ramamurthy, Gabriela Gibson-Lopez, Martin Goros, Jonathan A. Gelfond, Stephen Gelfond, Philip Balfanz, Melissa Deuter, Donald McGeary, Monica Verduzco-Gutierrez

**Affiliations:** ^1^South Texas Medical Scientist Training Program, UT Health San Antonio, San Antonio, TX, United States; ^2^Department of Pharmacology, UT Health San Antonio, San Antonio, TX, United States; ^3^Department of Rehabilitation Medicine, UT Health San Antonio, San Antonio, TX, United States; ^4^South Texas Psychiatry Practice-Based Research Network (PBRN), San Antonio, TX, United States; ^5^Department of Family and Community Medicine, UT Health San Antonio, San Antonio, TX, United States; ^6^Department of Population Health Sciences, UT Health San Antonio, San Antonio, TX, United States; ^7^Department of Psychiatry and Behavioral Health Sciences, UT Health San Antonio, San Antonio, TX, United States

**Keywords:** Long COVID, PTSD, stress, depression, anxiety, PCL-5, GAD-7, PHQ-9

## Abstract

Long COVID, previously known as Post-Acute Sequelae of SARS-CoV-2 (PASC), refers to prolonged symptoms or diagnosable conditions following COVID-19 infection. The neuropsychiatric profile of Long COVID patients remains ambiguous. This study aimed to assess neuropsychiatric symptoms in a retrospective cohort of Long COVID patients (*N* = 162) at a Rehabilitation Medicine clinic in South Texas. Clinical data from patient records were used to calculate a Symptom Score, and screening tools for stress/PTSD (PCL-5), depression (PHQ-9), anxiety (GAD-7), and quality of life (SWL) were employed to evaluate if Long COVID duration and severity could predict neuropsychiatric outcomes. The majority were female (71%) and Hispanics (53%) who presented for treatment of Long COVID symptoms during the study period, including fatigue (93%), coughing/shortness of breath (81%), fever (67%), anosmia (58%), ageusia (54%), and weight loss (56%). A minority of participants were hospitalized (*N* = 49) or required ventilator support (*N* = 5) during acute infection. There was a high burden of neuropsychiatric symptoms, including subjective cognitive impairment (79%), headache (74%), and insomnia (58%). Symptom Score (median = 9, IQR [8,11]) was significantly correlated with increased depression (PHQ-9; *p* < 0.05), anxiety (GAD-7; *p* < 0.05) and elevated stress/PTSD (PCL-5; *p* < 0.05) symptoms. Long COVID patients taking stimulants or mood stabilizers had higher GAD-7 (*p* < 0.031, *p* < 0.035) and PHQ-9 (*p* < 0.034, *p* < 0.009) scores but not PCL-5 scores. Importantly, duration of Long COVID symptomatology also did not predict PCL-5 scores. No patient factors (e.g., sex, age, BMI, ethnicity) mediated Symptom Score. Nonetheless, historically marginalized groups, such as women and Hispanics, have been disproportionately affected by COVID-19. This study is the first to utilize validated screening tools to determine the presence and severity of neuropsychiatric symptoms in Long COVID patients. These findings may guide clinical management and future research on Long COVID, especially in historically excluded populations.

## Introduction

The impact of the SARS-CoV-2 virus, which causes acute COVID-19 infection, is an ongoing public health concern in the United States (US) that is here to stay (1). Continued study of the multifaceted impact on health and longevity is essential to advance the development of effective therapeutic and prevention strategies. Most individuals recover from COVID-19 infection within 5-20 days, depending on severity of symptoms (2, 3); yet, a recent meta-analysis (4) estimated 31-69% of COVID-19 patients endure ongoing, relapsing and remitting, or progressive symptoms beyond 30 days of primary infection. The resulting syndrome is known as Post-Acute Sequelae of SARS-CoV-2 (PASC) (5) or Long COVID. Thus, Long COVID is now defined by the National Academy of Sciences, Engineering, and Medicine as an infection-associated chronic condition that occurs after SARS-CoV-2 infection and is present for at least 3 months as a continuous, relapsing and remitting, or progressive disease state (6).

Aggregated Long COVID symptomatology (5, 7–9) has been tracked in both small (10) and large (9, 11, 12) longitudinal cohort studies. In addition to continued symptoms of the acute infection (e.g., coughing, shortness of breath, loss of taste and smell), all studies to date have identified a common clustering of heterogenous symptoms affecting neuropsychiatric systems (4, 5, 9–12). These symptoms include chronic fatigue, cognitive impairment (“brain fog”), headache, pain syndromes, anxiety and depression.

Given the neurovirulent profile of SARS-CoV-2 (13), one might predict that the neuropsychiatric burden of Long COVID contributes significantly to complex treatment need and delayed recovery of affected patients. In fact, most patients experience reduced health-related quality of life (14) and Long COVID neuropsychiatric cluster symptoms for at least 6 months and upwards of 3 years out from primary infection (9). Thus, Long COVID poses a significant and chronic disruption to patient lives, as demonstrated by increased disability and economic burden reported in this population (15). Importantly, studies published to date have relied entirely on self-reported neuropsychiatric symptoms without the support of validated screening tools that could facilitate uncovering the etiology of neuropsychiatric symptoms. There is a high degree of clinical utility in defining quantifiable metrics to expediate diagnosis and treatment of the neuropsychiatric burden of Long COVID.

Known risk factors (9, 15–17) for Long COVID include those that predicate more severe COVID-19 infection, such as obesity, age, premorbid metabolic or cardiovascular conditions—and, critically, healthcare equity and access. Historically excluded groups in the US, like women and Hispanics, have been disproportionately impacted by the COVID-19 pandemic (15–20). A recent multi-site study following over 12,000 patients (73% female) found that female sex was significantly associated with higher risk of Long COVID (21). While there are currently no large cohort studies following Hispanics in the US, one study (22) sampling adults in Mexico found at least 5 persistent Long COVID symptoms in over half of participants (*N* = 192), with 360-day persistence probability of 0.78. Strikingly, the largest cohorts following the natural history of Long COVID in the US have disproportionately sampled Caucasian men (9, 11). Thus, there may be significant gaps in our understanding of how various patient factors may drive the complexity and duration of Long COVID symptoms.

There are no cures nor standard of care treatments for Long COVID apart from symptom management. Yet, as exemplified by emerging clinical trials (23, 24), there is considerable interest in investigating the reappropriation of existing pharmacotherapies, in addition to expanded immunization, for treatment and prevention of Long COVID (17, 25–28). While the results of these trials are highly anticipated, we may gather hints about under treated symptomatology in Long COVID patients by evaluating current pharmacologic profiles onboard at time of evaluation.

We hypothesized that while chronic stress and anxiety do not directly facilitate the development of Long COVID, the presence and severity of neuropsychiatric symptoms should track with the core symptomatology and drive symptom complexity. Thus, we sought to characterize Long COVID symptoms in a cohort of patients (*N* = 162; 71% female, 53% Hispanic, median BMI = 30 [obese]) who presented in a Rehabilitation Medicine clinic in South Texas. We applied a proof-of-concept methodology to rapidly screen patients for major clusters of Long COVID symptomatology (i.e., “Symptom Score”) to correlate symptom complexity with a battery of widely validated instruments for comorbid stress, anxiety, and mood disorders, as well as current medications, to measure their interactions with neuropsychiatric symptoms. We expected the results of this study to reveal any synergies with between neurovirulence and common neurobiological pathways associated to better inform the management of Long COVID patients, particularly in marginalized groups.

## Results

### Long COVID Symptom Score reflects neuropsychiatric complexities of symptomatology

**Figure 1:**
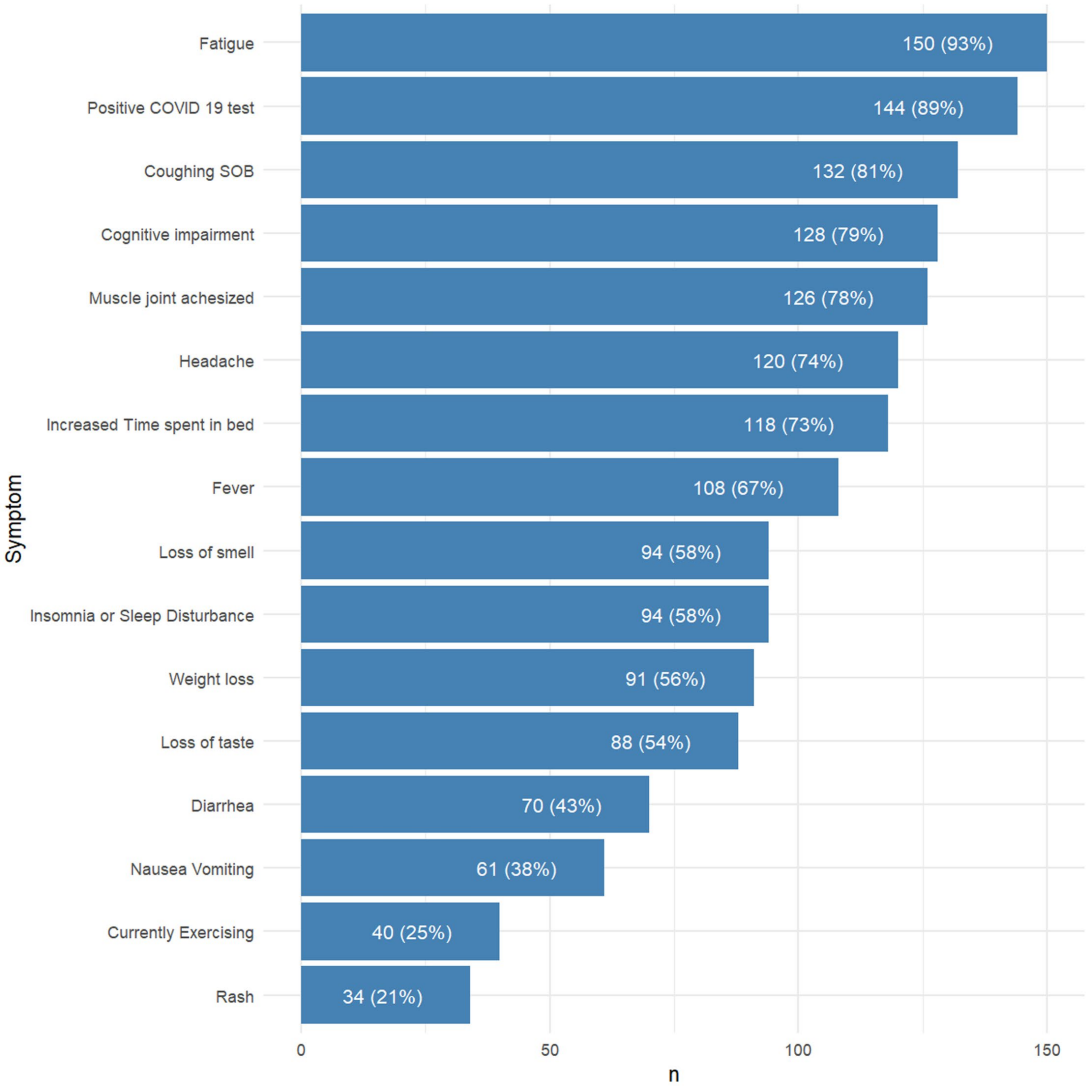
Summary of Symptom Score components and incidence (%) in analytical sample. We collected self-reported data on PASC symptomatology across all major systems as previously described (9, 11). Symptoms were ranked by incidence (% of patients who reported symptom) in our sample. We then calculated a PASC Symptom Score based on the total number of comorbid symptoms present in a patient based on the top 16 most common symptoms, summarized here. Of note, the most common PASC symptoms noted by our sample was fatigue (93%). There was a high burden of symptoms classically related to acute COVID-19 infection (coughing, shortness of breath [SOB], loss of smell and taste) as well as subjective neuropsychiatric symptoms (cognitive impairment = 79%, muscle/joint pain = 78%, insomnia/sleep disturbance = 58%).

We characterized the symptom profile in Long COVID patients (*N* = 162), defined as symptoms or conditions present for at least 30 days after acute COVID-19 infection. Importantly, 89% of the sample received an initial COVID-19 infection diagnosis by positive test (Figure 1). The distribution of approximate time elapsed since COVID-19 infection was approximately normal (Supplementary Figure S1). All patients received an initial COVID-19 diagnosis within one of the major peaks of the COVID-19 pandemic.

The 16 symptoms considered in this study were based on major categories determined by previous studies (5, 9, 11). The incidence-ranked symptoms used to calculate each participant’s Symptom Score within this cohort are summarized in Figure 1, where in the presence of each component was valued at 1 point, and cumulative symptoms are additive to the total score. Thus, a participant with a higher Symptom Score reflects a more complex symptomatology profile.

Notably, the mean Symptom Score in this cohort was 9 (IQR [8,11]; Table 1), indicating that at evaluation, the average participant reported more than half of the symptoms surveyed. The composition of participants’ Symptom Scores was heavily biased toward prolonged COVID-19 symptoms. The most common symptom reported in our cohort was fatigue (93%). Most patients reported a prolonged cluster of symptoms of acute COVID-19 infection, including coughing or shortness of breath (81%), fever (67%), loss of smell (58%), and loss of taste (54%).

**Table 1:** Summary of patient factors and prediction of long COVID Symptom Score.

Patient factor	Overall, *N* = 162^1^	PASC Symptom Score		*p*-value^2^
		> Median, *N* = 78^1^	≤ Median, *N* = 84^1^	
Symptom Score	9 (8, 11)	11 (10, 12)	8 (6, 8)	<0.001
Sex				0.2
Female	117 (73%)	60 (78%)	57 (68%)	
Male	44 (27%)	17 (22%)	27 (32%)	
(Missing)	1	1	0	
Age at evaluation	44 (35, 54)	44 (36, 52)	46 (34, 56)	0.4
BMI	30 (26, 36)	30 (26, 35)	29 (26, 36)	0.7
(Missing)	22	12	10	
Race/Ethnicity^3^				0.8
Black or African American	6 (3.9%)	4 (5.3%)	2 (2.6%)	
White	61 (40%)	29 (39%)	32 (42%)	
Hispanic	78 (51%)	38 (51%)	40 (52%)	
Other	7 (4.6%)	4 (5.3%)	3 (3.9%)	
(Missing)	10	3	7	
Time since infection (days)	190 (83, 278)	218 (83, 295)	169 (95, 264)	0.4
(Missing)	5	0	5	
Time since infection <30 days	8 (5.1%)	1 (1.3%)	7 (8.9%)	0.063
(Missing)	5	0	5	

1 Median (IQR); *N* (%).

2 Wilcoxon rank sum test; Pearson’s Chi-squared test; Fisher’s exact test (categorical variables).

3 Other includes: American Indian Alaska Native, Asian. Unknown set to Missing.

The contribution of a common cluster of neuropsychiatric symptoms to Symptom Scores in this cohort was striking. A substantial number of participants reported subjective cognitive impairment (79%), headache (74%), increased time spent in bed (73%), insomnia/sleep disturbance (58%). Many patients also reported new onset of muscle and/or joint pain (78%). Moreover, at the time of the assessment, only 25% were currently exercising.

Finally, symptoms commonly associated with Long COVID fatigue (29), such as gastrointestinal (diarrhea [43%], nausea/vomiting [38%], weight loss [58%]), and autoimmune cluster symptoms (rash [21%]) were reported. Though less common, the relative contribution of these symptoms to the overall burden of comorbidities could not be understated. Moreover, the presence of sparsely represented symptoms within the Symptom Score distribution especially prompted a need to determine if symptom complexity could be stratified by any patient factors to explain any etiologic vulnerabilities within specific populations.

### Canonical Long COVID patient risk factors do not stratify Long COVID Symptom Score

We next sought to determine if patient factors predicted Long COVID symptom burden (Table 1). We analyzed the relatedness of Symptom Score across the cohort to canonical, *a priori* patient factors selected based on current literature surrounding specific socioeconomic and health access vulnerabilities exacerbated during the COVID-19 pandemic (8–10, 18–20, 30–34). Additionally, we sought to determine if there were significant differences in patient factors between two subgroups stratified by the median Symptom Score. Symptoms Scores with “high” complexity were those over the median score (“> Median,” *N* = 78, mean Symptom Score = 11, IQR [10, 12]), while those with comparably “low” complexity were those equal to or less than the median score (“≤ Median,” *N* = 84, mean Symptom Score = 8, IQR [6, 8]). The mean Symptom Score of these two subgroups was significantly different (*p* < 0.001).

With these considerations, we correlated overall Symptom Score as well as membership to high or low symptom complexity subgroups with various patient factors, including biological sex, age, body mass index (BMI), race and ethnicity. More female (*N* = 117, 73%) than male (*N* = 44, 27%) patients presented for evaluation for Long COVID symptoms during the study period. There was a higher proportion of females with above median Symptom Score compared to males, but this difference did not reach statistical significance (78% vs. 68%, *p* = 0.2). Thus, despite significantly more females in our sample, there was no contribution of biological sex to Symptom Score.

Moreover, age (median = 44, IQR [35, 54]) and BMI (median = 30, IQR [26,36]) also failed to predict Symptom Score (*p* = 0.4, 0.7, respectively). Likely due to the geographic location of this study, most patients identified as Hispanic (53%). Yet, no race or ethnicity identified in this cohort were associated with Symptom Score (*p* = 0.8). Further, there was a high degree of balance between high and low Symptom Score complexity subgroupings, and no patient factor predicted membership to either subgroup.

Importantly, we noted that very few patients with Long COVID symptoms in this cohort reported being hospitalized (*N* = 49) or requiring a ventilator (*N* = 5) during initial COVID-19 infection. Moreover, few (N < 5) required oxygen therapy or sustained cardiac damage because of COVID-19 infection. Thus, Symptom Score composition in our cohort largely stems from a patient group with Long COVID symptomatology following non-severe primary COVID-19 infection.

### Multifaceted neuropsychiatric symptom clustering seen in Long COVID patients

Given the significant contribution of neuropsychiatric cluster symptoms to Symptom Score, we sought to determine if quantification of psychiatric symptoms could predict Symptom Score. In a first, we administered validated screening tools to assess the presence and severity of stress rising to the level of post-traumatic stress disorder (PTSD; PCL-5), anxiety (GAD-7), and depression (PHQ-9), and general satisfaction with life (SWL) to participants.

The result of relating the scores from these screening tools to Symptom Score are summarized in Table 2. PCL-5 administration revealed 44% of the cohort lived with stress-related symptoms that rose to the level of PTSD. GAD-7 scores revealed that 69% of participants with Long COVID demonstrated at least mild anxiety symptoms with 18% affected by severe anxiety. Most (74%) participants screened for depression with PHQ-9 had symptoms characterized by at least mild depression, with 28% experiencing moderately severe to severe symptoms. Approximately 41% of the cohort was slightly to extremely dissatisfied with life. One important nuance to the incidence of neuropsychiatric cluster symptoms in this and any cohort of Long COVID patients in the inability to reliably analyze this finding considering the index (inciting) event. Thus, despite strong evidence of neuropsychiatric burden, it was not possible to directly attribute the emergence of novel neuropsychiatric symptoms to Long COVID symptoms or COVID-19 infection.

**Table 2:** Categorical score results of screening tools for stress (PCL-5), anxiety (GAD-7), and depression (PHQ-9) symptoms, and satisfaction with life (SWL) in long COVID patients.

PCL-5 Score (*N* = 162)	*N* (%)
Not elevated	90 (56%)
Significantly elevated trauma symptoms of PTSD	72 (44%)
GAD-7 Score (*N* = 143)	***N* (%)**
No significant symptoms	44 (31%)
Mild	38 (27%)
Moderate	35 (24%)
Severe	26 (18%)
(Missing)	19
PHQ-9 Score (*N* = 146)	***N* (%)**
Minimal depression	38 (26%)
Mild depression	32 (22%)
Moderate depression	34 (23%)
Moderately severe depression	21 (14%)
Severe depression	21 (14%)
(Missing)	16
SWL Score (*N* = 147)	***N* (%)**
Dissatisfied	11 (7.5%)
Extremely dissatisfied	14 (9.5%)
Extremely satisfied	18 (12%)
Neutral	4 (2.7%)
Satisfied	39 (27%)
Slightly dissatisfied	35 (24%)
Slightly satisfied	26 (18%)
(Missing)	15

**Figure 2:**
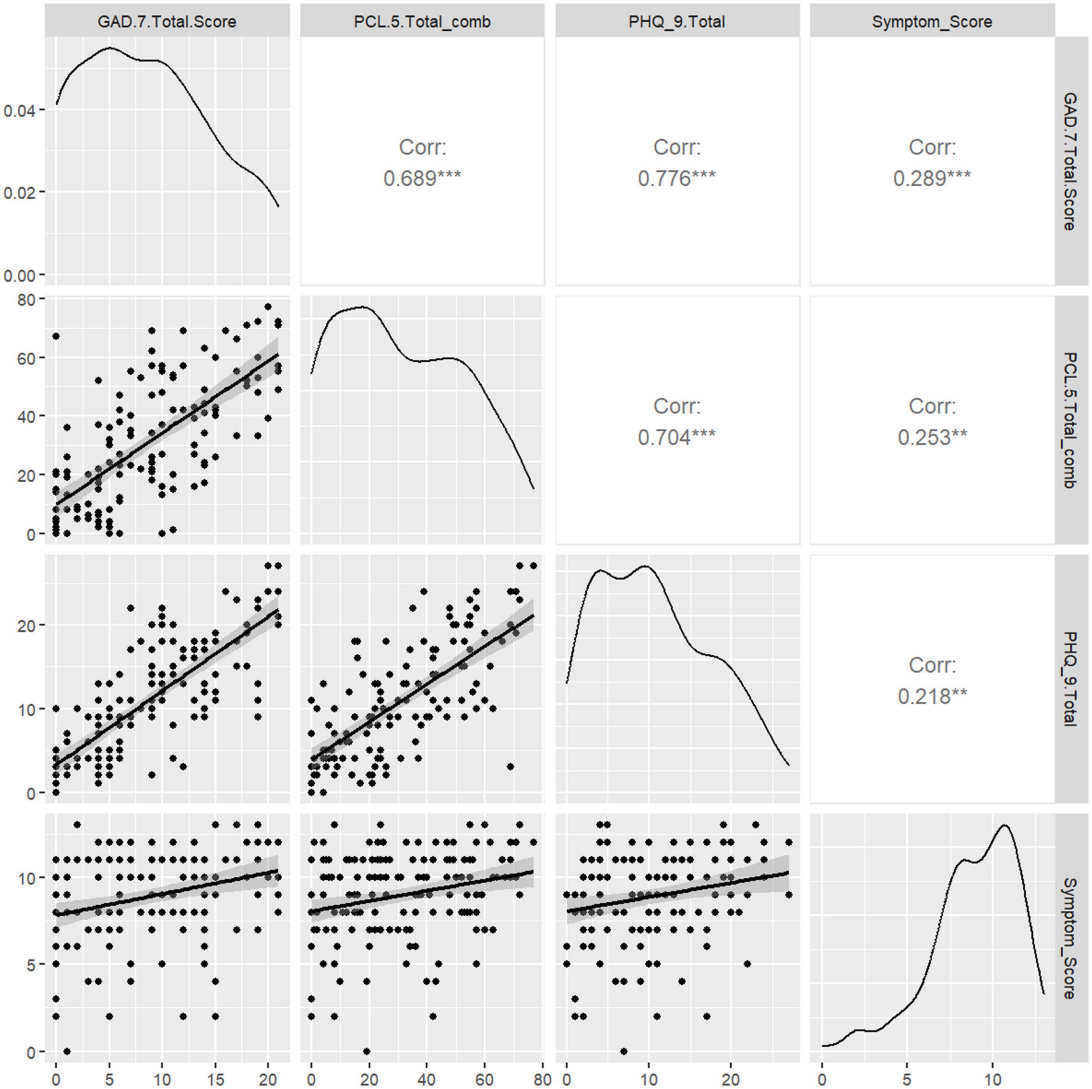
Correlation of Symptom Score with measures of depression (PHQ-9), stress (PCL-5), and anxiety (GAD-7) symptoms. We assessed whether PASC Symptom Scores were correlated with the presence of depression, stress, or anxiety symptomatology as determined by validated screening tools. Spearman rank correlations were computed for each pairwise combination of PASC Symptom Score and PCL-5 or GAD-7 scores. PHQ-9, PCL-5, and GAD-7 scores were mutually associated. PASC Symptom Score was positively associated with PHQ-9 (0.218, *p* < 0.05), GAD-7 (*r* = 0.29, *p* < 0.05) and PCL-5 (*r* = 0.25, *p* < 0.05).

We then sought to determine if Symptom Score informed stress and anxiety levels in this cohort by calculating Spearman rank correlations between each measure (Figure 2). PCL-5, GAD-7, and PHQ-9 scores were all mutually associated. Symptom Score was positively associated with PCL-5 (*r* = 0.25, *p* < 0.05), GAD-7 (*r* = 0.29, *p* < 0.05), and PHQ-9 (*r* = 0.25, *p* < 0.05) scores. Duration of Long COVID symptoms was not correlated with PCL-5, PHQ-9, or GAD-7 scores (Supplementary Figure S2). Thus, only complexity Long COVID symptomatology (e.g., worse stress, anxiety and depression symptoms) drove worse neuropsychiatric symptom status.

**Figure 3:**
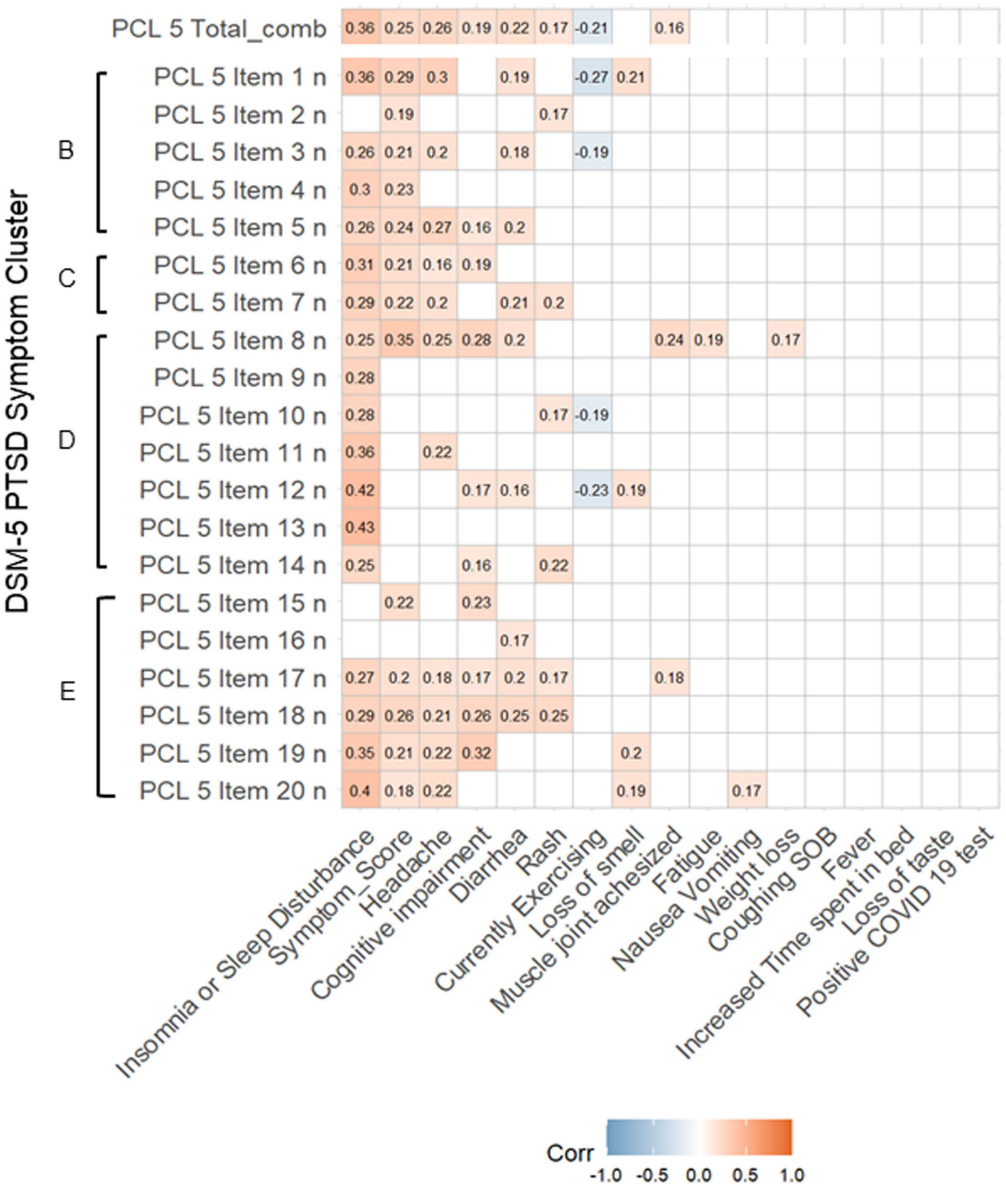
Correlation of individual PCL-5 items with Long COVID symptoms. Spearman rank correlations were calculated for the intersection of the score of each item, reflecting frequency of stress and PSTD-related symptoms by item of the PCL-5 and the PASC symptoms used to calculate PASC Symptom Score. We contextualize the PCL-5 item scores with their respective symptom clusters for Post-Traumatic Stress Disorder according to the DSM-5, designated to the left of each PCL-5 item: Cluster B (The traumatic event is persistently experienced), Cluster C (Avoidance of trauma-related stimuli after trauma), Cluster D (Negative thoughts or feelings began or worsened after the trauma), Cluster E (Trama-related arousal and reactivity that began or worsened after the trauma). Correlations are given for all findings with *p* < 0.05.

We partially refined this analysis to examine how each of the 20 items of the PCL-5 correlated with individual Long COVID symptoms that comprise the Symptom Score (Figure 3). Nearly all items on the PCL-5 were positively and significantly correlated with insomnia or sleep disturbance (17/20 items, *p* < 0.05). Importantly, individual item correlations do not vary by PTSD Symptom Cluster (B, C, D, E) as identified in the DSM-5. Headache (11/20 items, *p* < 0.05) and subjective cognitive impairment (9/20, *p* < 0.05) were also significantly positively correlated with many items. Of note, whether the patient was currently exercising at time of assessment was negatively correlated with 5 items (*p* < 0.05). The item most positively correlated with the greatest number of symptoms was item 8 (*p* < 0.05): “In the past month, how much were you bothered by: Trouble remembering important parts of the stressful experience?”

### Pharmacotherapy insufficient for managing neuropsychiatric symptoms with Long COVID

The complex symptom profile of Long COVID and high degree of association with neuropsychiatric burden for participants in this study begged the question: are Long COVID patients receiving adequate pharmacotherapy to manage symptoms? We suspected that a first approximation could be found by analyzing the impact of ongoing pharmacotherapies on neuropsychiatric symptoms.

To get a sense of broad patterns, we performed a limited scop analysis by reviewing classes of current prescriptions reported by each patient at the time of evaluation for Long COVID. Importantly, all medications reported were prescribed prior to the intake appointment without knowledge of prescription initiation or purpose of medication. Medications were operationalized as categorical variables organized by class designations. We then correlated PHQ-9, GAD-7 and PCL-5 scores with each class of medications at the time of Long COVID evaluation.

**Figure 4:**
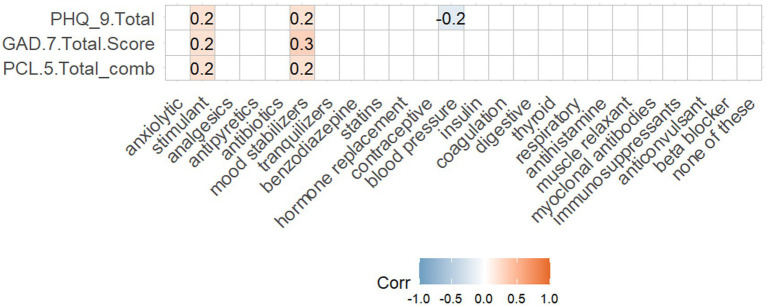
Correlation of depression (PHQ-9), anxiety (GAD-7), and stress (PCL-5) symptomatology with current medications. Spearman rank correlations were calculated for the intersection of HQ-9, GAD-7, and PCL-5 total scores with classes of current medications endorsed by self-report. Correlations are given for all findings with *p* < 0.05.

Intriguingly, higher PHQ-9, PCL-5 and GAD-7 scores were all positively correlated with prescriptions for mood stabilizers (e.g., valproate, carbamazepine, lamotrigine) and stimulant (e.g., methylphenidate, amphetamine/dextroamphetamine, modafinil, lisdexamfetamine) class medications (Figure 4), perhaps reflecting higher neuropsychiatric burden in patients already being treated for these symptoms. On the other hand, there was a negative but non-significant correlation between PHQ-9 score and blood pressure medication (Figure 4). No other class of medication evaluated in this study was significantly associated with scores on any neuropsychiatric instrument.

We sought to further this analysis beyond correlations. Participants who endorsed current prescriptions from mood stabilizer (*N* = 6) or stimulant (*N* = 8) classes of medications represented a small subset of the overall cohort (Table 3). When directly comparing this subset to the rest of the cohort, patients taking either class of medication had significantly higher GAD-7 scores compared to those not taking them (*p* [mood stabilizer] = 0.031, *p*[stimulant] = 0.035). A similar pattern was seen for PHQ-9 score (*p*[mood stabilizer] = 0.034, *p*[stimulant] = 0.009). Yet, there were no significant differences in PCL-5 scores between those prescribed either medication class or not. These results persisted despite potential for background of high variability with indication and duration of prescription prior to study intake.

**Table 3:** Correlation of anxiety, stress, and depression symptomatology with mood stabilizer and stimulant class medications.

Characteristic	Mood stabilizer class		Difference^2^	95% CI^2^^,^^3^	*p*-value^2^
	Yes, *N* = 6^1^	No, *N* = 156^1^			
GAD-7 Score	15.8 (6.3)	8.2 (6.0)	7.6	1.0, 14	0.031
(Missing)	0	19			
PCL-5 Score	54 (23)	30 (21)	24	-0.49, 48	0.053
PHQ-9 Score	18 (7)	10 (7)	7.9	0.89, 15	0.034
(Missing)	0	16			

Characteristic	Stimulant class		Difference^2^	95% CI^2^^,^^3^	*p*-value^2^
	Yes, *N* = 8^1^	No, *N* = 154^1^			
GAD-7 Score	13.1 (4.7)	8.3 (6.1)	4.9	0.46, 9.3	0.035
(Missing)	1	18			
PCL-5 Score	48 (23)	30 (21)	18	-1.7, 37	0.068
PHQ-9 Score	17 (5)	10 (7)	7.1	2.4, 12	0.009
(Missing)	1	15			

1 Mean (SD).

2 Welch two sample *t*-test.

3 CI, confidence interval.

Most participants endorsed at least one prescription from all other classes considered in this study (Supplementary Table S1). Of note, 41% of participants endorsed taking medications related to respiratory care (e.g., albuterol, salmeterol/fluticasone, fluticasone, tiotropium) well after initial COVID-19 infection. Given the limited scope of interview, we were unable to correlate endorsement of multiple prescriptions across classes (i.e., polypharmacy) with any other measure in this study.

## Discussion

Despite the best efforts of public health and medical systems, the COVID-19 pandemic remains a burden in much of the world, underscoring the need for further research into both the impact of acute infection and its associated infection-associated chronic condition, known as Long COVID (2–5)^.^ SARS-CoV-2, the virus that causes COVID-19 and thus Long COVID, is neurotropic (35–38) and thus propagates throughout the central nervous system. The symptom clusters experienced by both acute COVID-19 and Long COVID patients imply significant distortions to normal functioning of central nervous system due to neurovirulent damage. In this study, we present key findings and acknowledge limitations, aiming to encourage others to build on this work to elucidate the underlying mechanisms of Long COVID and drive the development of effective therapies and interventions.

### Symptom Score is an effective clinical tool for Long COVID evaluation

We characterized Long COVID symptoms in a cohort of patients who, due to the unique nature of our study site in South Texas, represented categories of patients thought to be a greatest risk (4, 16, 17, 20) for severe COVID-19 infection and thus Long COVID (*N* = 162; 71% female, 53% Hispanic, median BMI = 30). Additionally, in contrast to previous studies (9, 12, 20), few patients with Long COVID symptoms in our cohort were ever hospitalized (*N* = 49) or required ventilation (*N* = 5). Thus, while our cohort uniquely highlights the relative share of Long COVID patients presenting for treatment in this region, we emphasize discrete cohort composition differences that may drive differences between our results and prior studies. The largest studies tracking relatively homogenous Long COVID patient cohorts have implied that only patients with severe acute COVID-19 infection requiring hospitalization and supportive therapies like ventilators or oxygen supplementation (9, 11, 17, 22). Our study revealed that the risk for Long COVID extends far beyond severe infection, particularly for neuropsychiatric cluster of this syndrome. Thus, we reiterate the importance of inclusive recruitment efforts in Long COVID studies to capture the broad realities of Long COVID patient profiles, as well as redress the disproportionate burden faced by Long COVID patients with similar demographic profiles across the United States.

We used the Symptom Score to correlate complexity of multisystemic Long COVID symptomatology, which demonstrated a neuropsychiatric symptom cluster was highly consistent with larger and more detailed inventories of Long COVID symptoms from previous studies (9, 11). Although relatively abbreviated, our findings reveal a high burden of fatigue, cognitive impairment, and anxiety among Long COVID patients, exacerbating the core Long COVID symptom profile.

The significant impact on quality of life and the increased risk of worsening psychiatric disorders in Long COVID (31, 39) underscore the need for greater attention to neuropsychiatric symptoms in the management of Long COVID. Thus, we advance the Long COVID Symptom Score as an effective inventory or core symptoms across several major systems (e.g., cognitive, respiratory, neuropsychiatric, pain, and constitutive/gastrointestinal symptoms) for use in clinical settings that benefit from rapid screening tools. We encourage clinicians and researchers to validate the Symptom Score in diverse populations. The Symptom Score may also assist with triaging patients for additional tests to detect neurovascular changes (40), neuroinflammation (32, 41), and structural alterations (42–44) in patients with Long COVID.

### High neuropsychiatric burden may reveal specific vulnerabilities of CNS to COVID-19 infection

We hypothesized that chronic stress and mood disorders may significantly contribute to the complexity of Long COVID symptomatology, playing a key role in the neuropathophysiology associated with the condition. We found that comorbid stress and mood disorder symptomatology as measured by the PHQ-9, PCL-5 and GAD-7 instruments was significantly predictive of Long COVID symptomatology, over any other patient factor considered in this study. Our results are aligned with prior qualitative reporting on neuropsychiatric cluster enrichment in the Long COVID syndrome (9, 31, 32, 35, 36, 38, 45).

Due to the retrospective nature of this study, we are unable to assign causality to the pre-infection presence of neurocognitive, neurological, or neuropsychiatric symptoms to the Long COVID syndrome. However, the relative share of neuropsychiatric symptom burden to the average Long COVID patient profile may reflect a neuraxial vulnerability of the central nervous system to initial COVID-19 infection (12, 34, 37). Indeed, neuropsychiatric symptoms in Long COVID patients are known to persist for at least 3 years beyond initial infection (9). Nonetheless, the broad-spanning neuropsychiatric and cognitive burden of disease in this and other cohorts is more aligned with stochastic, diffuse neuropathological damage disrupting broad cognitive circuits (31, 36, 46–48); in contrast, a discrete pattern of damage would be expected to yield focal deficits.

Of course, one provocative alternative hypothesis is that validated instruments measuring neuropsychiatric symptoms coalesce on specific features Long COVID that overlap in neurobiological mechanisms that drive the same features of syndromic psychiatric disorders (31, 49–51). Our subanalysis correlating individual PCL-5 inventory items to Symptom Score components demonstrated the highest agreement between of just a handful of items with substantial cognitive and neuropsychiatric Long COVID symptoms. On the other hand, nearly all PCL-5 items were positive correlated with insomnia and sleep disturbance. One study demonstrated that insomnia is a common feature in the Long COVID profile, particularly in non-hospitalized patients (52). These results could indicate that a narrow list of brain regions or circuits impacted by a specific neuraxis of post-traumatic stress disorder are selectively vulnerable to COVID-19 infection. For example, the gatekeeping function of the locus coeruleus, which regulates sleep–wake cycles and is frequently disrupted in other neuropsychiatric conditions, may be persistently dysregulated in Long COVID patients (52–54). We suspect that similar patterns could be revealed by applying more refined and exhaustive neuropsychiatric and cognitive inventories to Long COVID patients (55–59).

One critical gap in Long COVID patient evaluation is access to an inventory to characterize symptoms related to the patient-reported cognitive impairment, which was endorsed by 73% of our cohort at time of assessment. Long COVID “brain fog” one of the most consistent and distressing syndrome features reported by patients (9, 11, 33, 35), and the cause is yet unknown. Recent studies point to sustained blood–brain barrier disruption and neuroinflammation in Long COVID patients to explain persistent cognitive impairments (32, 60, 61). Instruments used to assess cognitive impairment and dementia in other disease contexts (56, 57) with have not been extensively validated in the Long COVID patient population (62, 63). A standardized and validated tool to assess specific features of cognitive impairments in this and other populations could point to underlying neurobiological pathways or circuitry that are particularly vulnerable to neurovirulence of COVID-19 infection or chronic dysregulation leading to Long COVID.

### Pharmacotherapy indexing remains a gap in establishing Long COVID treatments

There is considerable interest in identifying effective therapeutics for treating or outright preventing its development (25–28). There is conflicting evidence that adequate management of pre-existing or comorbid conditions could mediate Long COVID burden on patients (9, 11, 31, 36, 64), to either protective or deleterious ends. While the cause of Long COVID syndrome following acute COVID-19 infection has yet to be established, there is some evidence that specific medication classes during acute phase confers elevated risk of Long COVID conversion (e.g., NSAIDs (64)).

Considering conflicting reports, we hypothesized that ongoing pharmacotherapy in participants with Long COVID symptoms could reveal patterns in the presence and severity of neuropsychiatric symptoms in our cohort. Our limited analysis highlights a subset of participants with the currently prescribed medications from classes typically associated with treating neuropsychiatric disorders (e.g., mood stabilizers, stimulants). We found some evidence supporting broad predictive value of medication classes with neuropsychiatric symptom burden in our cohort, albeit with small sample sizes.

As we await the results of the first clinical trials investigating a role for existing therapeutics for management of Long COVID (23, 24), our results offer a preliminary glance at what could be expected from larger studies. The reality is that Long COVID symptoms were complex and profound in all participants in this study, and no current medication fully addressed these symptoms. These results echo calls for novel approaches to Long COVID treatment that precisely address the underlying cause of the syndrome rather than stopping short at symptom management (25, 26, 28). We suspect our analysis ran against background variation in prescription initiation and duration prior to or concurrent with Long COVID onset. Nonetheless, we hope these results generate sufficient intrigue to merit study of pharmacotherapy as an index event for Long COVID for two motives: (1) to establish any additional risk or protective effects of pharmacotherapy classes for Long COVID symptom burden, or (2) to determine if management of neuropsychiatric comorbidities alleviates overall Long COVID burden.

### Study interpretations limited due to study setting

It is admittedly difficult to attribute stress, anxiety, or satisfaction with life measures specifically to COVID-19 infection or chronic Long COVID symptomatology without knowledge of any index or inciting traumatic events. We anticipate this may be a difficult confounding factor to address, given the general stress and anxiety levels induced by the global COVID-19 pandemic at the time of study recruitment. Given the retrospective study criteria and nature of the data collection, we are unable to temporally resolve the onset of Long COVID symptomatology with any measure considered in this study, including neuropsychiatric symptom burden as measured with validated instruments and the impact of pharmacotherapy on the same scales. We can neither confirm nor assume that all patients would have started prescriptions before initial COVID-19 infection, nor at some point along the development of Long COVID symptomatology. The latter would particularly impact patients with the greatest duration of Long COVID symptoms, as it leaves the greatest chance and window for seeking symptomatic treatment with any of the medication classes analyzed. Importantly, we also cannot rule out the impact of greater socioeconomic stressors experienced by historically marginalized groups on Long COVID Symptom Score, nor any measure of stress and anxiety used here. Our results reflect patient status captured within a single visit to a Rehabilitation Medicine clinic and thus would likely benefit from long-term follow-up with increased sample size.

## Materials and methods

This study was conducted under the approval of the Office of the Institutional Review Board at the University of Texas Health Science Center at San Antonio Long School of Medicine (protocol #20210194EX). Participants in this retrospective cohort study were men and women who were evaluated in a Rehabilitation Medicine outpatient clinic in South Texas from January 2020 to July 2021.

The study setting was an outpatient physiatry clinic in which each participant was evaluated, in a private room, by clinicians for Long COVID symptomatology and administered screening tools for stress, anxiety, and quality of life measures. The data was retrospectively collected directly from the medical chart in the University of Texas at San Antonio Health Science Center’s Electronic Medical Record (EPIC). Responses were recorded directly into RedCap and then aggregated in a de-identified database for statistical analysis.

### Analytical population

Eligible participants included patients over the age of 18 years old with a history of acute COVID-19 infection confirmed either by positive COVID-19 test (89%) or evaluation by a clinician for COVID-19 symptoms. Patients were seen at varying periods post-COVID-19 infection. Participants were ineligible if they were younger than 18 years old, did not have a history of acute COVID-19 infection, were evaluated for conditions apart from Long COVID, or were unable to read or speak in English to complete screening tools and clinical evaluation.

During the study period, 235 patients were clinically evaluated. In our analysis, we excluded patients who either did not complete the PTSD Checklist-Civilian Version 5 (PCL-5), Patient Health Questionnaire (PHQ-9), Generalized Anxiety Disorder Scale 7 (GAD-7), or Satisfaction with Life Scale (SWLS). Patients were not excluded from analysis if they were missing information on other patient factors or screening tools. We excluded these individuals from some sub-analyses (“Missing”), as noted throughout this report. The final analytical sample included 162 patients. These screening tools are outlined in detail below.

### Patient factors

In addition to the above outlined measures, we also collected patient factors for use as covariates in this study (Table 1). Patient factors included biological sex, ethnicity/race, age, education level, body mass index (BMI), pre-existing psychiatric disorders, current medication list, type of health care institution, region, and employment status.

### Long COVID Symptom Score

We collected self-reported Long COVID symptomatology noted in the medical record from a single clinical evaluation of Long COVID symptoms (9, 11) from all participants. Long COVID symptoms included: (1) positive COVID-19 test at the time of primary infection, (2) fatigue, (3) coughing or shortness of breath (SOB), (4) muscle or joint ache, (5) headache, (6) increased time spent in bed, (7) fever, (8) loss of smell, (9) loss of taste, (10) cognitive impairment, (11) insomnia or sleep disturbance, (12) weight loss, (13) diarrhea, (14) nausea or vomiting, (15) rash, (16) currently exercising. For our analysis, we then operationalized Long COVID symptoms and calculated a Symptom Score based on the total number of symptoms reported during clinical evaluation. We also evaluated factors thought to affect PASC symptom severity, such as the amount of elapsed time from Long COVID evaluation to initial acute COVID-19 infection, whether the patient was hospitalized, required ventilation or supplemental oxygen, or experienced cardiac damage because of primary infection.

### Stress and posttraumatic stress disorder (PTSD) assessment (PCL-5)

Posttraumatic stress disorder (PTSD) Check List for DSM-5 (PCL-5 (50, 65)) was used to assess stress and trauma-related symptoms. The PCL-5 was adapted for the Diagnostic and Statistical Manual of Mental Disorders-Fifth Edition (DSM-5) and demonstrated strong reliability and validity (49). The PCL-5 is a 20-item inventory designed to gage symptoms based on 4 major PTSD symptom clusters: Cluster B (The traumatic event is persistently experienced), Cluster C (Avoidance of trauma-related stimuli after trauma), Cluster D (Negative thoughts or feelings began or worsened after the trauma), Cluster E (Trama-related arousal and reactivity that began or worsened after the trauma). Participants rated the degree to which they experienced each symptom on a scale ranging from 1 (not at all) to 5 (extremely), with possible scores ranging from 17 to 85. Cut scores range from 30 to 60 depending on the variation and base rate of the disorder in the population and settings, and it is recommended to use higher cut-offs in populations with higher base rates, such as veterans, and lower cut scores with populations of lower base rates of PTSD (66). A total score of 31-33 suggests a diagnosis of PTSD and that the patient may benefit from PTSD treatment.

### Anxiety assessment (GAD-7)

We used the Generalized Anxiety Disorder-7 (GAD-7 (67)) to evaluate patients for presence and severity of anxiety. The GAD-7 is a self-report seven item questionnaire assessing symptoms of generalized anxiety disorder that has been found to have validity as a measure of anxiety in the general population (68). In this study, the GAD-7 was altered to reflect symptomatology within the time frame of the past month to match the frame of reference for other screening tools used in this study. GAD-7 scores were used to assign patients into standard categories based on anxiety-related symptoms: 0-4 = minimal anxiety, 5-9 = mild anxiety, 10-14 = moderate anxiety, 15-21 = severe anxiety.

### Depression assessment (PHQ-9)

We used the 9-item Patient Health Questionnaire (PHQ-9 (69)) to evaluate patients for presence and severity of depression. The PHQ-9 has a cut score of 6 that has been recommended for depression screening in primary care and a score of 10 or higher is used to detect symptoms of major depressive disorder (70).

### Satisfaction with life assessment (SWL)

The Satisfaction with Life Scale (SWL (71)) is a five item self-report Likert scale (“strongly disagree” to “strongly agree”) to measure satisfaction with life as a proxy for subjective well-being. Past studies have established adequate reliability and predictive validity in a wide range of age groups (72). We analyzed patients who fell within categorical score ranges.

### Statistical analysis

We used open-source statistical software (R version 4.3+, Vienna, Austria) to complete all statistical analyses in this study. Patient factors were tested for associations with Symptom Score by stratifying participants as above or below the median Symptom Score and using the Wilcoxon rank sum test (continuous variables) or Fisher’s exact test (categorical variables). Medication use for each category was used as a predictor within linear regression models for each outcome (PCL-5, GAD-7, PHQ-9, and Symptom Score). Spearman’s correlations were used to identify associations among these outcomes. All testing was two-sided (*α* = 0.05).

## Data Availability

The raw data supporting the conclusions of this article will be made available by the authors, without undue reservation.
