# Supplementary material for: Neuropsychiatric symptoms cluster as primary drivers of Long COVID complexity: a South Texas retrospective cohort study
Source: Front Neurol. 2025 Jul 23;16:1612489. doi: 10.3389/fneur.2025.1612489 (PMC12325076; doi:10.3389/fneur.2025.1612489)
Supplement: Supplementary file 1 [file Supplementary_file_1.docx]

**SUPPLEMENTAL MATERIAL**

**Neuropsychiatric Symptoms Cluster as Primary Drivers of Long COVID Complexity: A South Texas Retrospective Cohort Study**


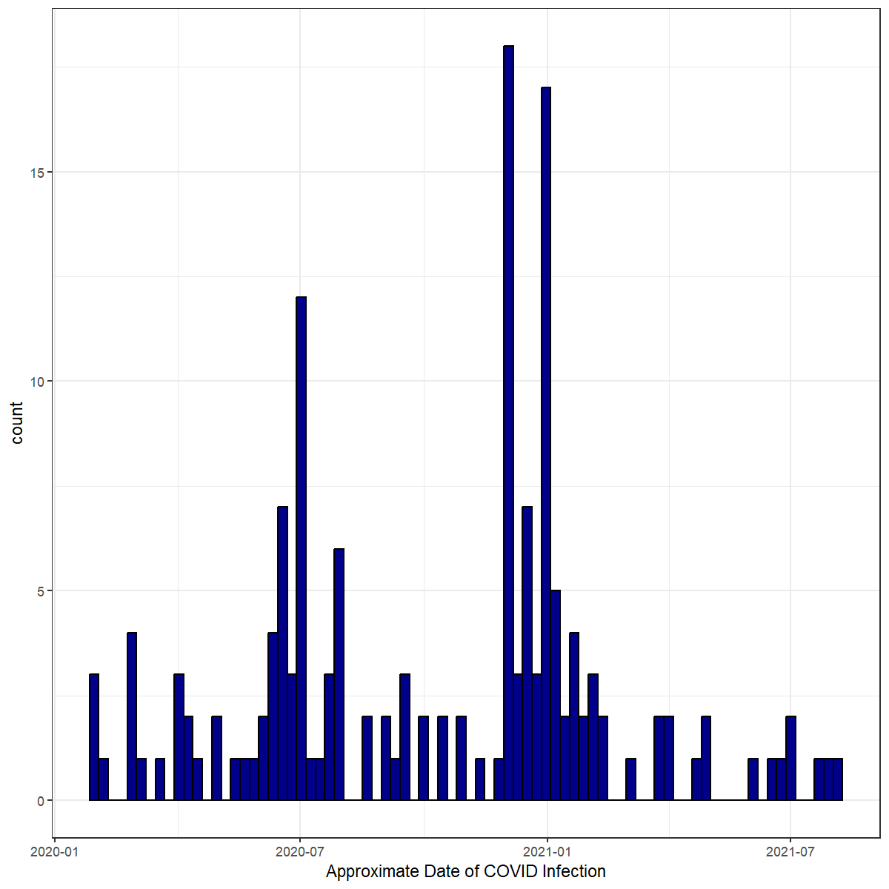


**Figure S1. Onset of initial COVID-19 infection in analytical sample aligns with major waves of pandemic.** We plotted the approximate date of initial COVID-19 infection in our patient sample and compared the distribution of peaks with data from local (Bexar County) and national (United States) peak rates of infection during the same time points. Distribution of onset in our cohort centers around the two major peaks (2020-07, 2021-01) of contemporaneous local and national COVID-19 infection rates.


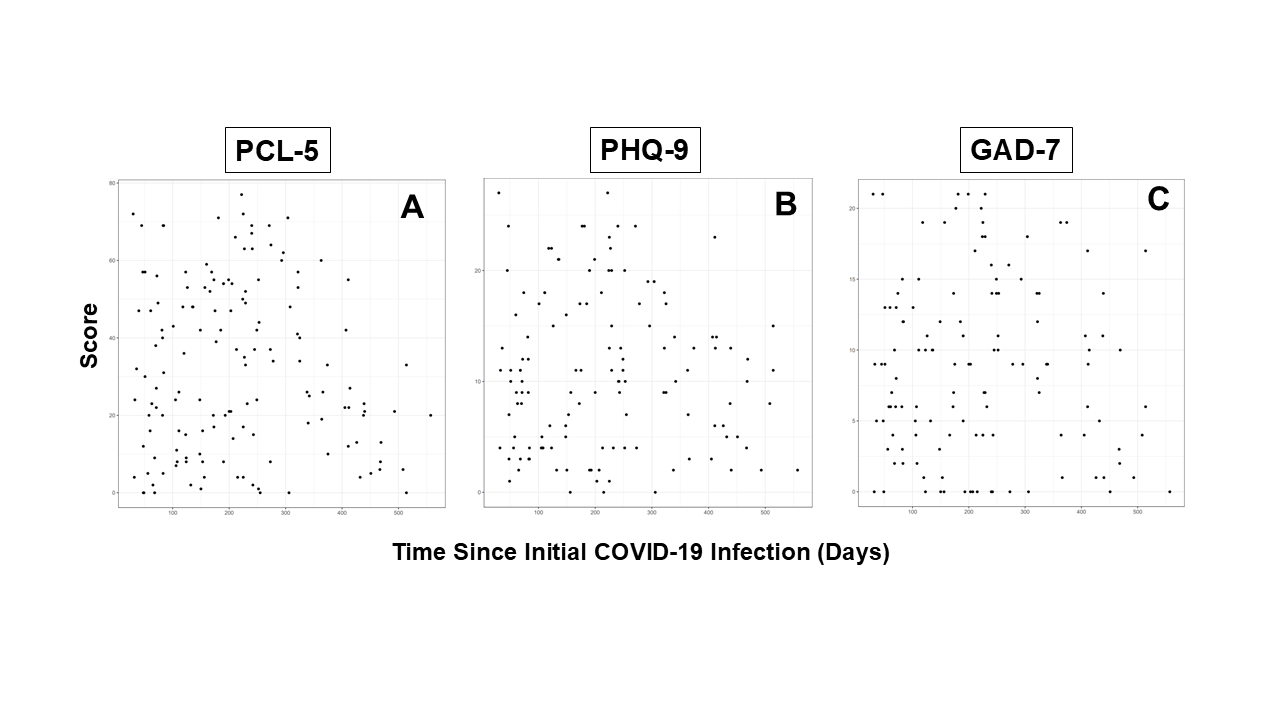


**Figure S2. Duration of Long COVID symptoms is not correlated with scores on stress/PTSD, depression, or anxiety symptom instruments.** Scatterplots of the correlation between time since initial COVID-19 infection (days) and: (A) stress/PTSD symptoms [PCL-5], (B) depression symptoms [PHQ-9], and (C) anxiety symptoms [GAD-7]. Screening tool scores (y-axis) are scaled relative to the score range of each inventory. There was no correlation between time elapsed since initial infection and any screening instrument for neuropsychiatric symptoms used in this study.

**Table S1: Counts of current medications reported by patients at time of Long COVID evaluation.**

| **Medication Class** | **N = 162***^1^* |
| --- | --- |
| **respiratory** | 66 (41%) |
| **anxiolytic** | 55 (34%) |
| **analgesics** | 51 (31%) |
| **antihistamine** | 43 (27%) |
| **digestive** | 40 (25%) |
| **blood pressure** | 33 (20%) |
| **statins** | 25 (15%) |
| **none of these** | 19 (12%) |
| **thyroid** | 18 (11%) |
| **insulin** | 17 (10%) |
| **beta blocker** | 16 (9.9%) |
| **muscle relaxant** | 15 (9.3%) |
| **hormone replacement** | 14 (8.6%) |
| **anticonvulsant** | 11 (6.8%) |
| **coagulation** | 11 (6.8%) |
| **antipyretics** | 10 (6.2%) |
| **benzodiazepine** | 9 (5.6%) |
| **immunosuppressants** | 9 (5.6%) |
| **contraceptive** | 8 (4.9%) |
| **stimulant** | 8 (4.9%) |
| **antibiotics** | 7 (4.3%) |
| **mood stabilizers** | 6 (3.7%) |
| **tranquilizers** | 5 (3.1%) |
| **myoclonal antibodies** | 2 (1.2%) |
| **none of these** | 19 (12%) |
| *^1^* N (%) | |
